# Mercury Toxicity and Neurogenesis in the Mammalian Brain

**DOI:** 10.3390/ijms22147520

**Published:** 2021-07-14

**Authors:** Louise C. Abbott, Fikru Nigussie

**Affiliations:** 1 Department of Veterinary Integrative Biosciences, College of Veterinary Medicine and Biomedical Sciences, Texas A&M University, 4458 TAMU, College Station, TX 77843-4458, USA; 2 College of Veterinary Medicine, Oregon State University, 700 SW 30th Street, Corvallis, OR 97331, USA; Fikru.Nigussie@oregonstate.edu

**Keywords:** neural stem cell, neural progenitor cell, developing neurons, methylmercury, developmental neurotoxicology, proliferation, migration, differentiation

## Abstract

The mammalian brain is formed from billions of cells that include a wide array of neuronal and glial subtypes. Neural progenitor cells give rise to the vast majority of these cells during embryonic, fetal, and early postnatal developmental periods. The process of embryonic neurogenesis includes proliferation, differentiation, migration, the programmed death of some newly formed cells, and the final integration of differentiated neurons into neural networks. Adult neurogenesis also occurs in the mammalian brain, but adult neurogenesis is beyond the scope of this review. Developing embryonic neurons are particularly susceptible to neurotoxicants and especially mercury toxicity. This review focused on observations concerning how mercury, and in particular, methylmercury, affects neurogenesis in the developing mammalian brain. We summarized information on models used to study developmental mercury toxicity, theories of pathogenesis, and treatments that could be used to reduce the toxic effects of mercury on developing neurons.

## 1. Introduction

### 1.1. Neurogenesis during Development

Neurogenesis is the process that produces neurons in the mammalian brain. A wide range of different types of neurons must be generated to form the functional brain. In mammals, these neurons are primarily formed during the embryonic, fetal, and early postnatal stages of development, but neurogenesis does continue throughout life in specialized regions of the adult brain. Considerable research has been devoted to neurogenesis that occurs in regions of the adult brain, especially in the mammalian hippocampal dentate gyrus. Increases in neural stem cell proliferation in the adult dentate gyrus in mammals has been called “reactive neurogenesis [1]”.

In this review, we have focused on neurogenesis that occurs in the embryonic mammalian brain. Neurogenesis in the developing mammalian brain has been most intensively studied and is best understood in the neocortex [2]. For that reason, we will provide a brief summary of normal neurogenesis that focuses on cortical development before we turn to the toxic effects of mercury exposure on neurogenesis.

**Figure 1:**
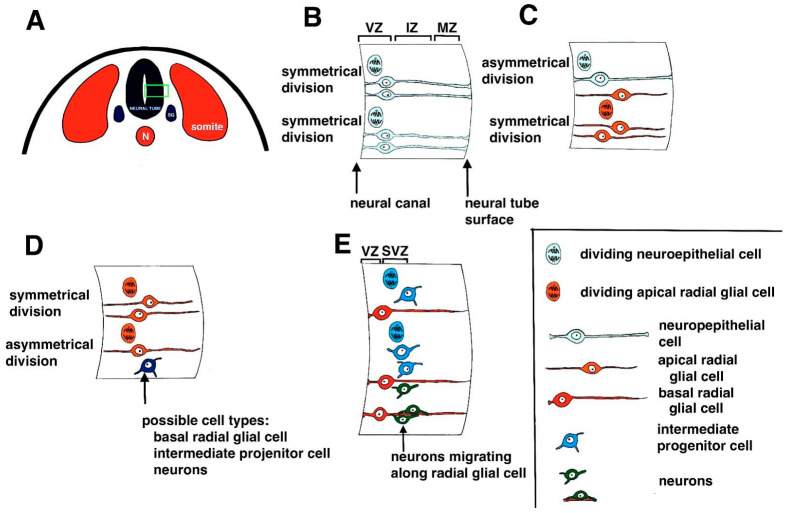
**Summary of neurogenesis**. (**A**) Schematic diagram of neural tube formation in a cross section of a mammalian embryo. The neural tube forms between paired somites. Below the neural tube is the notochord (**N**). During neural tube formation, neural crest cells migrate away from the neural plate to form numerous structures, including spinal ganglia (**SG**). The **green box** shows the area of the neural tube that is enlarged in diagrams B through E. (**B**) Early cell divisions in the developing neural tube. Neuroepithelial cells undergo symmetrical cell divisions to form more neuroepithelial cells. Cell divisions occur in the ventricular zone (**VZ**) located next to the neural canal. The mantle zone (**MZ**) or mantle layer is the layer of the neural tube next to the surface of the neural tube. In between the VZ and the MZ is the intermediate zone (**IZ**) or intermediate layer. (**C**) Neuroepithelial cells also give rise to apical radial glial cells that undergo symmetrical divisions to produce more apical radial glial cells. (**D**) Apical radial glial cells also undergo asymmetrical cell divisions to produce a range of cell types, including basal radial glial cells, intermediate progenitor cells, and early neurons. (**E**) Intermediate progenitor cells are located in a second germinal layer called the subventricular zone (**SVZ**) and undergo additional cell divisions. Intermediate progenitor cells give rise to neurons that migrate to their final locations in the developing central nervous system. The key seen in the bottom right corner of Figure 1 shows the different cell types described in the figure.

The original precursor cells that give rise to neurons are neural progenitor cells and stem cells [3,4,5]. The first neural progenitor cells to be formed in mammals are neuroepithelial cells (NECs) located in the inner ventricular zone of the developing neural tube, which lines the hollow space in the neural tube called the central (neural) canal (Figure 1A). These cells undergo numerous rounds of symmetrical cell division to increase the available progenitor cells (Figure 1B) [6]. Eventually, NECs give rise to pluripotent neural stem cells (NSCs) and apical radial glial cells (aRGs; Figure 1C) [3]. The aRGs undergo further symmetrical cell divisions to produce additional aRGs or asymmetrical division to give rise to additional cell types, including basal radial glia, intermediate progenitor cells, and neurons (Figure 1D) [6,7,8].

Intermediate progenitor cells (IPCs) (also called basal progenitor cells or BPs [9,10,11], are located in a second germinal layer called the subventricular zone and undergo additional cell divisions (Figure 1E) [12]. It has been noted that divisions of the aRGs cannot wholly account for the numbers of neurons found in the neocortex of higher mammals, including humans [12,13], and the IPCs are essential in the process of producing enough neurons to populate the developing cortex. Thus, IPC numbers are increased in species with an enlarged neocortex [14,15]. The IPCs eventually give rise to early neurons, which migrate to their appropriate destinations in the developing layers of the cortex (Figure 1E) [6,16].

**Figure 2:**
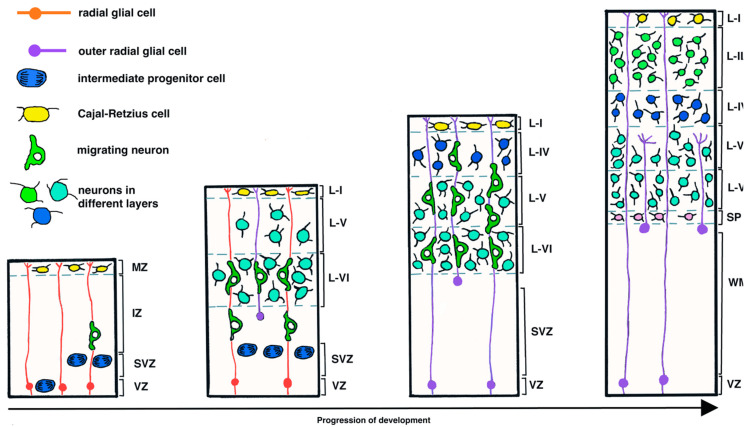
**Development of the six layers of the mammalian neocortex**. Figure 2 is a set of diagrams that show four different points during the development of the mammalian neocortex. The first diagram on the left is the earliest, and the last diagram on the right is the oldest. Neurons are initially formed in the ventricular zone (**VZ**) and then in the subventricular zone (**SVZ**). The earliest layers of the developing neural tube that gives rise to the central nervous system, which consists of the brain and spinal cord, are the VZ, intermediate zone (**IZ**), or intermediate layer, and the mantle zone (MZ) or mantle layer. Newly formed neurons migrate along radial glial cells to form Layers VI and V (**L-VI**, **L-V**) in an inside-to-outside pattern. Layer I (**L-I**) is an exception and forms separately. Subsequently, Layer IV (**L-IV**) is formed, and then Layers II and III (**L-II/III**). The different colors of neurons merely denote the different layers of neurons that form. Deep in L-VI, a region called the subplate (**SP**) forms, and the region of the SVZ develops into a layer of myelinated axons (white matter, **WM**). Note, this diagram does not show gliogenesis, which is initiated during the later stages of layer formation in the neocortex.

The developing neurons become organized into six specific layers in the cortex that are formed in an inside-to-outside pattern. Thus Layers VI and V are the first to form, then Layers IV, III, and II are formed sequentially (Figure 2) [17]. The exception to this pattern is Layer I. The cortical neurons found in Layer I are formed earlier than the neurons in the other layers [18]. The terms assigned to the different layers include Layer I as the molecular layer, Layer II as the external granular layer, Layer III as the external pyramidal layer, Layer IV as the internal granular layer, Layer V as the internal pyramidal layer, and Layer VI as the multiform/fusiform layer (Figure 2) [6]. Layers I and II/III are critical sites of connections that allow the integration of corticocortical inputs across cortical regions as well as the cerebral hemispheres [19,20]. Thalamocortical connections are mainly located in Layer IV, while Layers V and VI serve to connect subcortical regions with the cortex [6].

Numerous signaling molecules and pathways are involved in neurogenesis (see [6,21] for reviews). Some of the major pathways and molecules that have been described in detail include the Wnt/b-catenin signaling pathway, fibroblast growth factor ligands, bone morphogenetic proteins, sonic hedgehog, notch signaling, the roundabout (Robo) family of receptors and their ligands (Slit proteins), reelin, brain-derived neurotrophic factor (BDNF), phospholipase D1, and numerous transcription factors [6,21,22].

Some of the radial glial cells that are formed in early development become quiescent as the brain continues to develop and eventually form postnatal and adult NSCs in the subventricular zone (SVZ), located along the edges of the lateral ventricles [23,24]. Neuroepithelial cells in the embryonic hippocampal dentate gyrus migrate to the developing subgranular zone (SGZ) of the dentate gyrus, where they settle down to become quiescent cells that give rise to adult NSCS.

For this review, we restricted our comments to neurogenesis that occurs prenatally in mammals. However, a large body of literature has focused on the development of neurons occurring during adult neurogenesis. Therefore, the topic of how methylmercury exposure impacts adult neurogenesis was beyond the scope of this review, and we have not included a discussion of references that only covered adult neurogenesis. Instead, we have focused specifically on how mercury, and predominantly methylmercury, affects embryonic neurogenesis in mammals.

In the developing embryonic mammalian brain, neurogenesis occurs before gliogenesis [25]. Interestingly, the process of astrogenesis overlaps neurogenesis in the developing hippocampal dentate gyrus, which makes the process of neurogenesis and astrogenesis more complex in that brain region. [25] On the other hand, the effects of mercury toxicity are not influenced by astrocytes and oligodendrocytes during neural tube formation and early brain development.

During neurogenesis, specific modes of gene expression control the temporal sequence of transcription factor expression, including *Pax6*, *Ngn2*, *Tb2*, *TBr1*, *Ctip2*, and *Satb2*, that is necessary for differentiated, functional neurons to be formed [26,27]. For example, it has been observed in the early development of cortical neurons that *Tbr2* repressed the action of *Pax6* to initiate neuronal differentiation by activating *Tbr1*, *Ctop2*, and *SatB* [2,3,28].

### 1.2. Mercury in the Environment

Mercury is a highly toxic heavy metal present at low levels throughout the global environment, and the accumulation of mercury is due to natural and anthropogenic sources [29]. The United States Government Agency for Toxic Substances and Disease Registry (ATSDR) has ranked mercury (Hg) as the third most toxic element for human health [30]. Table 1 summaries mercury measured in whole blood, cord blood, and breast milk in various populations around the globe. This survey underscores the ubiquitous exposure that occurs.

After entering the environment due to natural and anthropogenic sources, mercury cycles between air, soil, and water [32]. Mercury that enters into bodies of water is methylated via sulfate-reducing bacteria residing in aquatic sediments to produce methylmercury [33,34,35]. Methylmercury is exceedingly toxic and easily crosses through cell membranes. Mercury toxicity in general exhibits a wide range of adverse effects, including inhibition of sulfhydryl-containing enzymes, which are critical components of cellular metabolism [36], increased reactive oxygen species (ROS) production, and lipid peroxidation [37], and disruption of intracellular calcium ion homeostasis [38,39].

Exposure to mercury is well known to affect the developing central nervous system (CNS) severely. Neural stem cell (NSC) progenitors and developing neurons, in particular, are extremely sensitive to heavy metal toxicity, which exerts numerous adverse effects on biological processes [35]. Much of the research into mechanisms by which mercury exerts its toxic effects in the CNS has focused on oxidative stress and apoptotic processes. With enough exposure, metal-induced cytotoxicity, degeneration, and apoptosis severely impair the developing CNS [40,41,42]. In vitro studies have revealed dose-dependent adverse effects in neuronal cell patterning and migration [43,44]. However, much of the published research on the neurotoxicity of mercury has focused on its effects on differentiated neurons and not NECs and NPCs.

This review focused on the specific effects of mercury toxicity on neurogenesis in the developing brain. Although the pathogenesis and underlying specific effects of mercury exposure on adult neurogenesis undoubtedly overlap with neurogenesis in the developing brain, the effects of mercury toxicity on adult neurogenesis are beyond the scope of this review.

## 2. Models Used to Study Developmental Mercury Toxicity

A number of models, both in vitro and in vivo, have been used to investigate the possible mechanisms underlying the pathogenesis of mercury’s toxic effects on neurogenesis [43,44,45,46,47,48]. Faustman et al. [49] used both in vivo assessment of mouse brains developmentally exposed to methylmercury and in vitro culture of primary neuroepithelial cells derived from the midbrain of fetal rats (gestational day 12). In vitro culture of NSCs allows assessment of the adverse effects of neurotoxicants on critical neurodevelopmental processes, and the ability of NSCs to proliferative provides a viable system to study mitotically inherited effects in vitro [50]. NSCs show higher sensitivity to a range of toxicants compared to mature neurons and glial cells [51]. Therefore, NSCs are a relevant in vitro model for toxicity assessment.

It is well-accepted that gene/environment interactions are critical in the developing CNS but studying such interactions is nearly impossible with only in vivo modeling. The use of in vitro models such as those based on induced pluripotent stem cells and, recently, whole genome analysis coupled with in vitro models have provided more advanced and rapid methods to assess developmental neurotoxicity. One particular advantage of using induced pluripotent stem cells to study developmental neurotoxicity is that their differentiation in vitro parallels known brain development stages that occur in utero [52]. Whole genome analysis has been applied to assess gene expression in cell lines exposed to neurotoxicants, including methylmercury [53]. However, the use of defined sets of mRNA biomarkers instead of the whole genome has proven to be more affordable and can make the screening of substances more economical [53].

Non-mammalian models also have been used to study neurotoxicity. In particular, the nematode *Caenorhabditis elegans* has been used extensively to study mechanisms of neurodegeneration [54]. Several previous studies have demonstrated that exposure to methylmercury causes changes in dopaminergic neurons [55,56]. *C. elegans* also has been used to study how methylmercury affects the cholinergic and monoaminergic systems [57].

## 3. Theories of the Pathogenesis of Mercury Toxicity in Neurogenesis

It is well known that mercury, particularly methylmercury, exhibits greater neurotoxicity in the developing embryonic nervous system compared to mature neurons [47,49,53,58]. Normal brain development and function are dependent on a coordinated balance of a range of cellular processes during development, including proliferation, differentiation, and cell death [44,59]. Research has demonstrated that immature neurons and neuronal precursors present a specialized or unique susceptibility to mercury toxicity [51]. While this phenomenon has been studied for many years, the underlying mechanisms of pathogenesis still are not clearly delineated. Available data suggest that multiple mechanisms are likely to be involved in how mercury adversely affects neurogenesis. The effects of mercury, and in particular, methylmercury, on cell signaling that results in disruption of cell proliferation, appear to be central to mercury toxicity rather than increased cell death [48,49,59]. However, it is clear that exposure to higher concentrations of mercury does result in the death of neuronal precursor cells as well as mature neurons, typically through the process of apoptosis [59].

### 3.1. Disruption of Cell Proliferation

Changes in NSC proliferation typically result in decreased numbers of neurons and microcephaly [60,61]. The likely molecular mechanisms by which exposure to low mercury concentrations disrupt cell proliferation include inhibition of DNA synthesis, alterations in gene expression, increased oxidative stress, altered protein phosphorylation, and disruptions in intracellular calcium ion (Ca^2+^) homeostasis [49,50,62].

Methylmercury has been shown to inhibit DNA synthesis and reduce levels of cyclins D1, D3, and E in developing neurons as well as CDK2, suggesting that methylmercury toxicity is due at least in part to preventing the transition from G1 to S during mitosis [59,63]. Additional studies have shown that methylmercury exposure impaired cell proliferation by affecting p16 and p21 levels [49,50]; p16 and p21 are cyclin-dependent kinase inhibitors that inhibit the cell cycle G1 to S-phase transition, which results in cell cycle arrest [50]. Faustman et al. [49] also reported that the elevated expression of several genes involved in cell cycle control and growth arrest, including two *GADD* genes, *GADD45* and *GADD153*. *GADD* genes also act at the G1 to S checkpoint [49]. It was interesting to note that progression through the cell cycle was not completely inhibited by exposure to methylmercury in the Faustman et al. [49] study, which was considered to be due to redundancy in the pathways regulating the cell cycle [64].

Yuan et al. [48] observed that with inhibition of proliferation caused by exposure to extremely low sub-nanomolar methylmercury concentrations, primary culture cortical precursor cells also exhibited dose-dependent, increased differentiation into neurons. On the other hand, differentiation was decreased when the precursor cells were exposed to higher concentrations of methylmercury, which is similar to previous reports in the literature [65,66]. One possible mechanism for these observed effects could be due to the effects of methylmercury that reduce intracellular glutathione (GSH) levels [48]. Neuronal differentiation of NSCs occurs when the GSH/glutathione disulfide (GSSG) ratio decreases [67]. Notch signaling also has been proposed as a mechanism by which methylmercury could inhibit neuronal differentiation [68]. Similar effects have been reported for low concentration exposure of methylmercury in dissociated human progenitor cells [69], human cell-derived neurospheres [70], and mouse embryonic NSCs [71].

### 3.2. Disruption of Gene Expression, Cell Signaling Pathways, and Protein Phosphorylation

Numerous studies have reported that methylmercury adversely affects cell signaling cascades that control the neural progenitor cell differentiation into astrocytes [68,72]. Neural progenitors are induced to form astrocytes primarily by activating the JAK/STAT signaling pathway [73,74]. Methylmercury enhances JAK/STAT responsive gene expression, which can shift neural progenitor cell differentiation towards gliogenesis [75]. Thus, the formation of increased numbers of glial cells at the expense of neuron formation could adversely affect brain formation and function. Microarray datasets have been used to examine alterations in gene expression that were induced in developing embryonic mouse neurons that had been exposed to a low level of mercury [76]. In that study, it was reported that the expression of GABA receptors was altered in response to mercury exposure; specifically, GABRA3 and GABRA6. They conclude that these receptors could be used as biomarkers for embryonic neuronal developmental neurotoxicity.

Methylmercury also alters protein phosphorylation, as reported by Jebbett et al. [75]. They found exposure to quite low methylmercury concentrations enhanced ciliary neurotrophic factor (CNTF)-induced STAT3 phosphorylation, while exposure to higher concentrations reduced phosphorylation. Additional evidence that methylmercury alters intracellular protein phosphorylation comes from studies by Monroe and Halvorsen [77] and Xu et al. [63]. In particular, exposure to low concentrations of methylmercury has been shown to inhibit ERK1/2 phosphorylation [63]. Thus, inhibition of proliferation and induction of cell cycle arrest in NSCs could be due in part to disruption of the ERK1/2 cell signaling pathway.

MicroRNAs (miRNAs) are known to be central players in the epigenetic regulation of neurogenesis, neuronal differentiation, and neurite outgrowth [78,79]. Nerini-Molteni et al. [78] analyzed miRNA expression profiles using cultured human pluripotent cells and identified significant changes in the expression of 12 miRNAs. Pallocca et al. [79] examined changes in miRNA expression caused by methylmercury exposure of pluripotent carcinoma stem cells and human embryonic stem cells at the stage of neural progenitor commitment to neurons. They concluded that several miRNAs that were involved in regulating essential developmental processes showed altered expression after the cells were exposed to methylmercury. Therefore, it is promising that analysis of miRNA expression could help assess developmental neurotoxicity and assist in predictive testing [79].

### 3.3. Oxidative Stress

Reactive oxygen species (ROS) primarily are considered to be deleterious in the developing and mature CNS. However, recent reports have assessed the abilities of ROS, such as superoxide, to act as signaling molecules in normal cell physiology [75,80]. Notwithstanding, the generation of excessive oxidative stress or reducing cellular oxidative defense capacity due to exposure to neurotoxicants, such as mercury, exerts deleterious effects on neurogenesis [51]. As expected, in a study that exposed cultured NPCs to methylmercury, higher exposure concentrations enhanced superoxide production [75]. Interestingly, exposure to very low methylmercury concentrations enhanced STAT3 signaling [75], supporting a positive role for ROS signaling in neurogenesis.

### 3.4. Disruptions in Intracellular Calcium Ion (Ca^2+^) Homeostasis

Because Ca^2+^s display a myriad of functions in cellular metabolism, perturbation of intracellular Ca^2+^ levels has been identified as a primary mechanism contributing to mercury neurotoxicity [51,81,82]. Some of the known effects include altered ability to mobilize Ca^2+^ from intracellular stores and altered levels of Ca^2+^ entry through calcium membrane channels [51,81,83]. Other effects include actions of Ca^2+^ as a second messenger, including actions on inositol phosphate levels and altering protein phosphorylation [84]. Inevitably, a complex interaction occurs between mechanisms as well. For example, it has been reported that Ca^2+^ is released from mitochondria when they are exposed to oxidative stress [51]. Thus, increased oxidative stress due to mercury exposure can affect cell function directly and indirectly through perturbations of Ca^2+^ homeostasis. It also is known that increases in intracellular Ca^2+^ levels can activate calcium-dependent enzymes and alter protein kinase functions [49].

### 3.5. Disruptions in Migration

Due to the high affinity of methylmercury for thiol groups, numerous targets exist in cells [43]. However, several lines of evidence suggest that exposure to methylmercury during neurogenesis in humans resulted in aberrant cell migration and disorganized neocortical layering [60] as well in animal models [85]. It has been proposed that methylmercury redirects genetic programs during neural development by altering cell signaling events to disrupt patterning and normal cell migration, resulting in dysplasia and abnormal cortical cytoarchitecture [43,60,61]. An essential signaling pathway in neural development and patterning that is disrupted by exposure to methylmercury is the Notch receptor pathway [86,87]. Using cultured neurons, methylmercury has been shown to inhibit cell migration profoundly [88]. Supporting evidence comes from the ability of methylmercury to disrupt the polymerization of microtubules, which is a mechanism by which cell migration and format of the mitotic spindle can be inhibited [49,89].

### 3.6. Long-Lasting Effects of Mercury Exposure

Investigators have determined whether exposure to methylmercury has a long-lasting effect on proliferation (mitotic inheritance) using in vitro models. Bose et al. [50] used cultured NSCs exposed to nanomolar methylmercury concentrations to answer this question. The NSCs that were directly exposed to methylmercury (P1 cells), as expected, showed significant inhibition of proliferation. More importantly, daughter cells (D2 and D3 cells) derived from P1 cells, which were never directly exposed to methylmercury, also showed inhibited cell proliferation. Similar to what was observed for P1 cells, p16 and p21 genes that inhibit cyclins and cyclin-dependent kinases also were upregulated in the D2 and D3 cells [50]. In addition, the D2 and D3 cells exhibited decreased phosphorylation of ERK1/2, which is an essential component in the G1/s phase transition in mitosis [90]. These changes were the effects described above on the daughter cells of P1 methylmercury-exposed cells, and were accompanied by decreased global methylation [50,91], which strongly indicated that epigenetic changes might play critical roles in the adverse effects of methylmercury exposure on neurogenesis.

## 4. Possible Treatments to Reduce the Toxic Effects of Mercury on Developing Neurons

As this review highlights, some progress has been made to increase our understanding of the pathogenesis underlying mercury toxicity and neurogenesis. However, relatively little research has been published on possible therapies that can reverse or diminish the toxic effect of mercury on developing neurons. One study by Falluel-Morel et al. [92] tested the effects of N-acetyl cysteine (NAC) for protective effects on mercury exposure in the developing brain. NAC is derived from L-cysteine and is used clinically to treat forms of drug intoxication and metal toxicity, including promoting methylmercury excretion in urine in adults [92,93]. In their study, Falluel-Morel et al. [92] noted that NAC administration prevented deleterious effects of acute methylmercury exposure to developing neurons in vitro and in vivo. Specifically, NAC prevented reductions in DNA synthesis and reversed increases in cell death caused by exposure to micromolar concentrations of methylmercury.

Another study examined the possible therapeutic effects of a leaf extract of *Dendropanax morbifera (*DML*)*, which has been reported to have various biological functions in the nervous system. DML is a plant found in South Korea and is used in traditional folk medicine to treat headaches [94]. Another species of *Dendropanax*, *D. morbiferawas*, has been reported to ameliorate neuronal damage when tested in an animal model of Parkinson’s disease [95]. DML significantly reversed the decreased rate of neuronal proliferation induced by methylmercury exposure in the rat hippocampus [96]. Furthermore, administration of DML significantly reversed reductions in proliferating cells and differentiated neuroblasts that resulted from administering methylmercury in an in vivo rat model [96].

A third study examined the therapeutic effects of an extract of *Lycium bararum* polysaccharides (LBPs) on methylmercury-induced damage in hippocampus NSCs [97]. LBPs appear to interact with numerous targets and have multiple pharmacological effects, including protection of the nervous system and repair of damaged neurons [98,99]. Administration of LBPs to NSCs exposed to methylmercury ameliorated the adverse effects of methylmercury on the development of exposed NSCs [97]. Specifically, LBP administration reduced the rate of abnormal differentiation and alleviated damage to NSCs caused by MeHg. Thus, these indicated that LBPs also might be an effective therapy to counter reduced neurogenesis caused by MeHg.

Additional reports have indicated that docosahexaenoic acid (DHA) may have positive effects on mercury neurotoxicity [100,101]. It is known that DHA is an essential component for neural development, including neurogenesis and neurite formation [102,103]. Thus, it is possible that treating mercury neurotoxicity with DHA during neurogenesis could be beneficial. However, much of the research with DHA as a possible treatment for mercury neurotoxicity has focused on its effects during adult neurogenesis and the progression of neurodegenerative diseases such as Alzheimer’s disease.

Concerning the use of non-mammalian models in which possible treatment has been assessed for mercury intoxication in developing organisms, one study examined the protective effects of guarana (*Paullinia cupana*) on developmental delay in *C. elegans* associated with mercury exposure [104]. *Paullinia cupana* Mart var. *sorbilis* is a plant native to Brazil and other parts of the Amazon basin. [105]. Arentes et al. [104] reported that exposure to guarana reduced the developmental delay that occurred with methylmercury exposure, which was likely due to a range of effects, including upregulating metal transport genes, detoxification, and the antioxidant properties of the compound.

## 5. Limitations

The focus of this review was to provide an updated understanding of the mechanisms that are involved in the toxic effects of mercury on embryonic neurogenesis in mammals. However, new information has been relatively limited in the past few years. Much of the current research has focused on adult neurogenesis. It is the case that mechanisms are likely to overlap between the effects of embryonic neurogenesis and adult neurogenesis. However, few studies have investigated the possible overlap. Additionally, while some progress has been made concerning viable treatments that could successfully ameliorate mercury toxicity in the developing nervous system of the mammalian embryo, additional research is needed. Thus, we suggest that future research focus on possible treatments.

## 6. Conclusions

**Figure 3:**
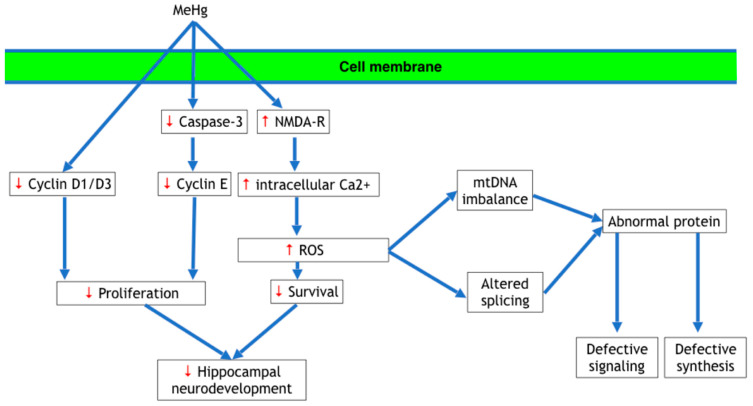
Summary of the neurotoxic effects of methylmercury during neurogenesis. This diagram summarizes the neurotoxic effects of methylmercury after it enters into developing cells, including neuroepithelial cells, intermediate progenitor cells, and migrating and differentiating neurons. Abbreviations: MeHg, methylmercury; NMDA-R, NMDA receptors; Ca^2+^, calcium ion; ROS, reactive oxygen species; mtDNA, mitochondrial DNA. Sources used to create this summary include references [38,42,59,64,84,106].

Neurogenesis is a complex process involving many steps and produces a vast array of different neuronal and glial subtypes. Based on the information reviewed above, it is evident that mercury has profound neurotoxic effects on neurogenesis. Thus, mercury toxicity is of concern to the global population. Much of the research on the neurotoxic effects of mercury has focused on methylmercury exposure, which is appropriate as methylmercury readily passes into tissues and enters cells. Numerous reports have documented that neural stem cells, the central cells of neurogenesis, are highly vulnerable to methylmercury. Considering the range of intracellular mechanisms affected by methylmercury exposure, diverse cellular models are needed to identify the various neurotoxic effects and the molecular mechanisms underlying mercury toxicity. Currently, the predominant theories of the pathogenesis of mercury toxicity in neurogenesis fall into the following three categories: (1) disruption of cell proliferation, gene expression, cell signaling pathways, protein phosphorylation, and calcium ion homeostasis; (2) production of oxidative stress; (3) altered cell migration. These effects of mercury, and in particular, methylmercury, are summarized in Figure 3. This review highlights the fact that these adverse effects are interconnected in complex ways. For example, alterations in calcium ion homeostasis and protein phosphorylation disrupt microtubule formation, which inhibits normal cell migration. Changes in gene expression affect cell proliferation. Future research should focus on combined investigations of changes in molecular effects such as gene expression and signal transduction with biochemical processes such as calcium ion homeostasis and cellular effects, such as cell proliferation.

Finally, little progress has been made on developing treatments to reduce the toxic effects of mercury on developing neurons. A few compounds have been studied for their possible therapeutic effects, but no clinical applications are on the horizon. Therefore, because mercury and especially methylmercury are ubiquitous in our environment, it is imperative to reduce exposure of the developing embryo.

## Figures and Tables

**Table 1:** Worldwide measurements of mercury: A, in whole blood (μg/L); B, in cord blood (μg/L); C, in breast milk (μg/L). These data were extracted from the article by Sharma et al. [31].

	A	B	C
North America			
USA	0–0.5	1–2.5	4–10 (Alaska)
Canada	2.5–4	4–5.8	0.2–0.5
Mexico	0.5–2.5		1–1.7
South America			
Brazil	10–30	10–30	4–10
Peru	30–108		
Chile	5.8–10		
Colombia	4–5.8		
Venezuela	10–30		
Ecuador	5.8–10		
Suriname	5.8–10		
Europe	0–0.5		
Italy	5.8–10	2.5–4	0.5–1
Belgium	5.8–10	5.8–10	
Spain	5.8–10	5.8–10	
Germany			1.7–3
Sweden			1.7–3
United Kingdom	2.5–4		
Denmark (Greenland)	10–30	30–53.3	
Finland	5.8–10		
Asia	5.8–10		
Russia	0.5–2.5		
China	2.5–4	2.5–4	1–1.7
India	30–108		4–10
Japan	5.8–10	10–30	0.5–1
Indonesia	5.8–10		3–4
Philippines	5.8–10	30–53.3	1.7–3
Singapore	30–108	30–53.3	
Turkey	0–0.5	0–0.5	10–30
Iran	2.5–4	1–2.5	2.5–4
Africa			
Egypt	10–30		
Nigeria	0.5–2.5	4–5.8	4–10
Benin	2.5–4		
Ghana	30–108		4–10
Zimbabwe	5.8–10		
Morocco	4–5.8		
South Africa	0–0.5	0–0.5	

## Data Availability

Not applicable.
